# Robust Control Strategy of Acoustic Micro Robots Based on Fuzzy System

**DOI:** 10.3390/mi15111403

**Published:** 2024-11-20

**Authors:** Junjie Dong, Xingguang Duan

**Affiliations:** School of Mechanical Engineering, Beijing Institute of Technology, Beijing 100081, China

**Keywords:** micro robots, fuzzy system, acoustic

## Abstract

This study presents a robust control strategy for acoustic micro robots utilizing a novel interval type-three fuzzy system. Micro robots driven by acoustic forces face significant challenges in fluid environments due to complex nonlinearities, uncertainties, and disturbances. To address these issues, we propose a control framework that combines fuzzy logic and sliding mode control to enhance the stability and trajectory tracking performance of micro robots under varying fluid conditions. The interval type-3 fuzzy logic system provides increased robustness by better handling external disturbances and uncertainties compared to the robustness of the traditional methods. The experimental results from one-dimensional, two-dimensional, and three-dimensional fluid cavities demonstrate that the proposed control method significantly improves tracking accuracy, reducing the errors in complex environments. This control framework offers promising potential for the precise manipulation of micro robots in biomedical applications and other microfluidic systems. The minimum trajectory tracking control mean square error is 12.82 μm.

## 1. Introduction

Ultrasound is an effective way to control micro robots because it can penetrate tissues, is unaffected by the opaque nature of biological bodies, and produces a wide range of forces. However, the navigation capability of ultrasonic micro–nano robots is poor, and the numerous parameters involved in their movement make it highly challenging for the conventional control methods to accurately control their movement in real time. Therefore, fuzzy control strategies are employed to accurately identify micro–nano robots and manipulate them using ultrasound for motion control. This paper demonstrates the robust motion control of ultrasonic micro–nano robots in fluid environments. The propulsion strategy relies on the combined effect of acoustic radiation forces, which guide the micro–nano robots along the desired trajectory. The fuzzy system control method for micro–nano robots has been validated, showing good robustness and providing effective control strategies that enable them to navigate independently in unstructured environments without external assistance. Inspired by the helical geometry of spirochete bacteria, an acoustically driven helical micro–nano robot has been proposed to operate in high-viscosity environments utilizing fin-like double-helix blades for movement. This micro–nano robot responds to external acoustic stimuli and mimics the helical motion of spirochetes and spirilla, which are natural micro–nano aquatic organisms. The asymmetric double-helix shape interacts with the incident acoustic field to generate a propulsive torque, causing the micro–nano robot to rotate about its long axis. Additionally, the proposed micro–nano robot has the unique feature of being able to switch propulsion direction simply by adjusting the frequency of sound waves.

A model-free control scheme for a dual-particle magnetic micro–nano robot system was proposed [1]. A proportional–integral (PI) controller was established for position control, and an extended state observer (ESO) was used for disturbance observation and compensation. Pawashe’s [2] team used a PID controller to implement the visual servoing of Mag-uBot for multimodal micromanipulation applications. In an in vitro scenario, the PID controller showed a good performance. Li and colleagues implemented the high-precision closed-loop steering of a vision-based micro–nano robot in zebrafish embryos [3]. The researchers proposed the use of PID for guidewire insertion regulation [4] and bending direction error attenuation. Magnetotactic bacteria (MTB) can migrate controllably based on the excitation of the external magnetic field. Khalil and colleagues [5] implemented the closed-loop control of MTB, where the bacteria tended to align themselves along the magnetic field direction, and the drive was controlled by its flagellar bundle and magnetic force. A micro–nano robot is a complex system with high nonlinearity and uncertainty. Unmodeled dynamics, Brownian motion [6], external fluid forces [7], and inaccuracies in the drive system [7] may all change the controllability of a micro–nano robot.

Adaptive control is applicable to systems without precise mathematical models. Researchers have applied adaptive controllers to microparticle manipulation [8,9] and cell tracking control for external disturbances and unknown dynamics [10]. When the system has large parameter perturbations and uncertainties, a well-designed, robust controller can guarantee an ideal performance. In the field of micro–nano robotics, input state stability (ISS) theory [11], sliding mode and backstepping control [12], and H-infinity control [13] are used to deal with disturbances and uncertainties in the working environment. In addition to adaptive control and robust control, Researchers have also proposed other nonlinear controllers to achieve the precise manipulation of micro–nano robotics. Reference [14] proposed a drive system capable of navigating a micro–nano robot in 3D space. A specified performance controller was proposed for motion control, and an observer was responsible for disturbance compensation. Reference [8] used a nonlinear backstepping controller combined with a high-gain observer to manipulate magnetic particles in the cardiovascular system to suppress the wall effect at the bifurcation of blood vessels. Reference [15] proposed a closed-loop control scheme for Caenorhabditis elegans using an optogenetic driving strategy. Navigation was based on a predictive proportional controller and could achieve micron-level accuracy. Reference [16] proposed a backstepping controller using a mean value theorem (MVT) observer to navigate a micro robot in a cylindrical container environment. The micro–nano robot could be navigated to move smoothly along a reference path under the influence of external nonlinear disturbances, especially hydrodynamic drag. Reference [9] proposed an agent-based sliding mode control strategy to achieve the 3D stable control of a spiral microswimmer. Ryan’s team developed a magnetic drive system consisting of eight permanent elements, each of which can rotate along a fixed axis [17]. A nonlinear path following algorithm was used to drive the micro–nano robot to reach the next path point, while approaching the path vertically. The acoustically driven micro–nano robot used various ciliary arrays to move the micro–nano robot through streaming. Researchers imitated the shape of starfish larvae and used a ciliary structure to generate water flow when oscillating and propelling the entire structure forward. C. Samson [18] proposed a method to linearize a nonlinear system into a chain form and designed a simple and effective controller for a two-wheeled robot system. This method was also used to manipulate a spiral microswimmer [19]. Reference [20] designed an enlarged spiral micro–nano underwater robot that tracks a planar path. The robot’s state model in the horizontal plane was rewritten as a linear system with three states and two inputs, and the desired direction was followed accordingly. Reference [21] proposed a bicycle model to describe the kinematics of a guidewire and used chain transformation to achieve model linearization and feedback controller design.

The rationale for using an interval type-three fuzzy system (IT3FS) in micro–nano robot control lies in its superior handling of uncertainties. Micro–nano robots operate at microscopic scales, where they encounter various sources of uncertainty, such as material properties and microscopic environmental disturbances. Unlike type-one and type-two fuzzy systems, the IT3FS incorporates an additional layer of uncertainty representation, enabling more accurate descriptions of complex system states and their relationships with inputs. Additionally, the IT3FS provides enhanced noise robustness, crucial for micro–nano robots that often operate in noisy environments, such as drug delivery in the body or precision surgical tasks, where random disturbances are frequent. The IT3FS’s multidimensional interval-based design helps to maintain control stability under such conditions. Furthermore, the IT3FS effectively meets the demands of complex control tasks, such as precise positioning and attitude control, through its advanced multivariable fuzzy mapping capabilities, significantly improving control accuracy. Despite its structural complexity, the IT3FS optimizes computational efficiency by constraining the uncertainty range, making it more feasible for real-time, low-power micro–nano robotic applications. Therefore, the IT3FS offers clear advantages in high-precision, high-robustness control under the challenging conditions typical of a micro–nano robotic environment [22,23,24].

The greatest characteristic of fuzzy control is that it does not require precise modeling; it specifies fuzzy rules based on prior experience and experimental data, making it a commonly used algorithm for addressing uncertainty. However, the control strategies for micro–nano robots are generally more complex because they must consider nonlinear effects in microscopic environments and dynamics at micro scales. Thus, it is essential to introduce uncertain systems for the motion control of micro–nano robots. Fuzzy control has developed over many years and has proven feasible for addressing uncertainty and complex dynamic control problems. Recently, interval type-three fuzzy logic systems (IT3FLSs) have been shown to estimate uncertainty and external disturbances more robustly. Because type-three fuzzy sets are defined as type-two fuzzy sets and type-one and type-two fuzzy sets are fixed-value and type-one fuzzy sets, interval type-three sets have more degrees of freedom. This approach can better reduce the tracking errors and improve the performance, making the IT3FLS more robust. The most common application of IT3FLSs is in controllers. Additionally, many other industrial fields utilize IT3FLSs, such as the event-triggered management of multi-agent systems, voltage management in battery systems, and stabilizing voltage converters, as well as data-driven gyroscope systems that incorporate IT3FLSs.

## 2. Controller Design

### 2.1. Kinetic Model

No matter what size the robot is in a liquid environment, it will be affected by the resistance of liquid. This paper uses a spiral robot. The robot experiences resistance from fluid in the acoustic field, represented by the secondary Bjerknes force between particles, and the propulsion force *F* is mainly provided by the primary Bjerknes force and the secondary Bjerknes force. The viscous resistance received in the liquid is expressed as

(1) $$F_{d}=6\pi \Omega av$$

where *a* is the radius of the robot’s section, Ω is the viscosity of the liquid, and *v* is the speed of the robot. The robot is subjected to various forces during movement, such as acoustic force, viscous resistance, gravity, buoyancy, and noise. However, the robot in this paper moves in a relatively closed cavity, so its dynamic equation can be written as

(2) $$M\ddot{O}=F-F_{d}+D$$

*M* is the mass of the robot. *D* is the interference term of the robot. *F* is the propulsion of the robot. Ö represents the displacement of the micro–nano robot. Thus, we have following state equation

(3) $$\left\{\begin{array}{l} {\dot{x}}_{1}=x_{2}\\ {\dot{x}}_{2}=\alpha u+\Pi \end{array}\right.$$

(4) $$\Pi =\frac{\beta }{M}\left(F_{d}-D\right)$$

(5) α=1/M

where *u* = *F* and α and β are positive parameters. $x_{1}$ and $x_{2}$ are displacement and velocity, respectively. Π is the sum of all the external unmodeled and external interference forces.

Microfluidic systems involve fluids passing through submillimeter channels under the influence of external forces. As the size decreases, the ratio of surface area to volume increases. At the micro–nano scale, surface tension and viscosity exceed gravity and inertia, which ensures the absence of turbulence and the occurrence of regular and predictable laminar flow. Ultrasonic manipulation occurs within the volume of fluid within the microfluidic channel. Due to the finite impedance of these solid boundaries, the pressure field in the fluid will inevitably cause the movement and deformation of the surrounding solids. The resonance and damping of the acoustic wave mode used in the fluid are affected by the neighboring materials, such as the piezoelectric ceramic transducers in excitation mode in the container and the glue that sticks them to the container. Classical ultrasonic transducers usually operate at resonant frequency, so it is important to avoid the overheating of transducers when using continuous mode, especially ceramic transducers, which exhibit this characteristic.

Micro–nano robots are subject to various disturbances in complex environments, which affect the motion control accuracy of micro–nano robots. To deal with disturbances in the path tracking control process of micro–nano robots, two aspects need to be considered:

(1) The real-time position of the micro–nano robot can be obtained using a camera, but the total disturbance cannot be directly measured, so a fuzzy system needs to be designed to estimate it.
(2) A controller must be designed that combines sliding mode control with the fuzzy system to eliminate the total disturbance, suppress the tracking error, and ensure the robustness of path tracking.

### 2.2. Fuzzy System

Now introduce the definition of an interval type-three fuzzy system. First, we define the following fuzzy set as a type-three fuzzy set, denoted as follows:

(6) $$A^{\left(3\right)}=\left\{\left(x,u(x),v(x,u),\mu_{A^{\left(3\right)}}(x,u,v)\right)|\;x\in X,u\in U\subseteq [0,1],v\in V\subseteq [0,1]\right\}$$

(7) $$\begin{array}{l} A^{\left(3\right)}=\int_{x\in X}\int_{u\in [0,1]}\int_{v\in [0,1]}\mu_{A^{\left(3\right)}}(x,u,v)/(x,u,v)\\ A^{\left(3\right)}=\int_{x\in X}\int_{u\in [0,1]}\int_{v\in [0,1]}\mu_{A^{\left(3\right)}}(x,u,v)dvdudx \end{array}$$

where *U* is the primary variable domain of *u*; *V* is the secondary variable domain of *v*.

(8) $$A^{\left(3\right)}=\int_{x\in X}\mu_{A_{x}^{\left(3\right)}}(u,v)/x$$

(9) $$\mu_{A_{x}^{\left(3\right)}}(v)=\int_{u\in [0,1]}\mu_{A_{\left(x,u\right)}^{\left(3\right)}}(v,u)/u$$

(10) $$\mu_{A_{\left(x,u\right)}^{\left(3\right)}}(x,u)=\int_{v\in [0,1]}\mu_{A^{\left(3\right)}}(x,u,v)dv$$

where $\mu_{A_{\left(x,u\right)}^{\left(3\right)}}$ is main membership, $\mu_{A_{x}^{\left(3\right)}}(u,v)$ is the main membership function, and $\mu_{A_{\left(x,u\right)}^{\left(3\right)}}\left(v\right)$ is the third-level membership function of the three-type fuzzy set. The following definition is given at this time:

(11) $$\mu_{A_{x}^{\left(3\right)}}(x,u,v)=1$$

Then, we obtain the interval type-three fuzzy set (IT3FS), which is defined as follows

(12) $$A=\int_{x\in X}\left[\int_{u\in [0,1]}\left[\int_{v\in \left[\underline{\mu }(x,u),{\bar{\mu }}_{A}(x,u)\right]}1/v\right]/u\right]/x$$

where

(13) $$\mu_{A(x,u)}(v)=\int_{v\in \left[{\underline{\mu }}_{A}\left(x,u\right),{\bar{\mu }}_{A}\left(x,u\right)\right]}1/v$$

(14) $$\mu_{A(x)}(u,v)=\int_{u\in [0,1]}\left[\int_{v\in \left[{\underline{\mu }}_{A}(x,u),{\bar{\mu }}_{A}(x,u)\right]}1/v\right]/u$$

(15) $$A=\int_{x\in X}\mu_{A(x)}(u,v)/x$$

$v\in \left[{\underline{\mu }}_{A}(x,u),{\bar{\mu }}_{A}(x,u)\right]$ is defined, where ${\underline{\mu }}_{A}(x,u)$ and ${\bar{\mu }}_{A}(x,u)$ are type-two membership functions. Following this, the following function ${\tilde{\mu }}_{A}\left(x,u\right)\in \left[{\underline{\mu }}_{A}(x,u),{\bar{\mu }}_{A}(x,u)\right]$ is defined, and then we obtain the interval type-three fuzzy membership function. The complete definition is as follows:

(16) $$A=\int_{x\in X}\int_{u\in [0,1]}\;{\tilde{\mu }}_{A}(x,u)/(x,u)$$

And because the type-two lower membership function belongs to the upper membership function, and ${\underline{\mu }}_{A}\left(x,u\right)\leq {\bar{\mu }}_{A}\left(x,u\right)$, an interval type-three fuzzy set consists of two interval type-two fuzzy sets, a type-two upper membership function and a type-two lower membership function. Then, we can obtain the following definition of interval type-three fuzzy sets:

(17) $$\underline{A}=\int_{x\in X}\int_{u\in [0,1]}{\bar{\mu }}_{A}(x,u)/(x,u)=\int_{x\in X}\left[\int_{u\in [0,1]}{\underline{f}}_{x}(u)/(u)\right]/x$$

(18) $$\bar{A}=\int_{x\in X}\int_{u\in [0,1]}{\bar{\mu }}_{A}(x,u)/(x,u)=\int_{x\in X}\left[\int_{u\in [0,1]}\bar{f_{x}}(u)/(u)\right]/x$$

The secondary membership functions are all type-one fuzzy sets, as shown below:

(19) $$\mu_{\underline{A}(x)}(u)=\int_{u\in J_{x}}{\underline{f}}_{x}(u)/u$$

(20) $$\mu_{\bar{A}(x)}(u)=\int_{u\in J_{x}}{\bar{f}}_{x}(u)/u$$

Alternatively, we can use another simple expression formula because each projection surface is an interval type-two membership function, and it can be written as follows:

(21) $${\tilde{f}}_{x^{\prime }}(u)\in \left[{\underline{f}}_{x^{\prime }}(u),{\bar{f}}_{x^{\prime }}(u)\right]$$

Thus, Formula (16) can be simplified to the following form:

(22) $$A=\int_{x\in X}\int_{u\in [0,1]}{\tilde{\mu }}_{A}(x,u)/(x,u)=\int_{x\in X}\left[\int_{u\in [0,1]}{\tilde{f}}_{x}(u)/(u)\right]/x$$

where ${\tilde{\mu }}_{A}(x,u)\in \left[{\underline{\mu }}_{-A}(x,u),{\bar{\mu }}_{A}(x,u)\right]$ or

(23) $$A=\int_{x\in X}\mu_{A(x)}(u)/x=\int_{x\in X}\left[\int_{u\in [0,1]}{\tilde{f}}_{x}(u)/(u)\right]/x$$

where $\mu_{A(x)}(u)$ is an interval type-two fuzzy set:

(24) $$\mu_{A(x)}(u)=\int_{u\in J_{x}}{\tilde{f}}_{x}(u)/u=\int_{u\in \left[{\underline{f}}_{x}(u),{\bar{f}}_{x}(u)\right]}1/u$$

The membership function of A is represented by ${\tilde{\mu }}_{A}(x,u)$, and the specific form is as follows:

(25) $$A=\left\{\left(\left(x,u),{\tilde{\mu }}_{A}(x,u\right)\right)|\;x\in X,u\in U\equiv [0,1]\right\}$$

where ${\tilde{\mu }}_{A}(x,u)\subseteq [0,1]$.

In this article, the triangular function is used as the membership function. The triangular function has three parameters on the uncertain coverage domain, namely $a_{1}$, $b_{1}$, and $c_{1}$. Its specific function form is as follows:

(26) $$\bar{\mu }(x)=\left\{\;\begin{array}{cc} 0 & x<a_{1}\\ \frac{x-a_{1}}{b_{1}-a_{1}} & a_{1}\leq x\leq b_{1}\\ \frac{c_{1}-x}{c_{1}-b_{1}} & b_{1}<x\leq c_{1}\\ 0 & x>c_{1} \end{array}\right.$$

The lower membership function of the uncertainty region in the vertical projection domain is determined by the coefficient $a_{2}$, $c_{2}$, which is determined by the coefficient $a_{1}$, $b_{1}$, $c_{1}$ of the upper membership function and another coefficient $l=\left(l_{1}+l_{2}\right)/2$. The specific formula is as follows:

(27) $$a_{2}=b_{1}-\left(b_{1}-a_{1}\right)\left(1-l_{1}\right)$$

(28) $$c_{2}=b_{1}+\left(c_{1}-b_{1}\right)\left(1-l_{2}\right)$$

(29) $$\mu (x)=\left\{\begin{array}{ll} 0 & x<a_{2}\\ \frac{x-a_{2}}{b_{1}-a_{2}} & a_{2}\leq x\leq b_{1}\\ \frac{c_{2}-x}{c_{2}-b_{1}} & b_{1}<x\leq c_{2}\\ 0 & x>c_{2} \end{array}\right.$$

Finally, the lower membership function can be obtained from (29). It only needs to be multiplied by the coefficient λ, which is defined as $\underline{\mu }(x)=\lambda \mu (x)$; thus, the upper membership function and the lower membership function are defined. Next, there are two more coefficients that affect the uncertainty area; one is the range and the other is the radius, which are defined as follows:

(30) $$\delta (u)=\bar{u}(x)-\underline{u}(x)$$

(31) $$\sigma_{u}=\frac{\delta (u)}{2\sqrt{3}}+\varepsilon$$

where ε is the calculation error. Then, the vertex or center of the three-type membership function of the entire interval can be expressed by the following formula:

(32) $$m(x)=\left\{\begin{array}{ll} 0 & x<a\\ \frac{x-a}{b_{1}-a} & a\leq x\leq b_{1}\\ \frac{c-x}{c-b_{1}} & b_{1}<x\leq c\\ 0 & x>c \end{array}\right.$$

$a=\left(a_{1}+a_{2}\right)/2$, $c=\left(c_{1}+c_{2}\right)/2$, so we can summarize the above conclusions to derive the expression of the interval type-three membership function:

(33) $${\bar{\mu }}_{A(x)}(u)=\exp[-\frac{1}{2}{\left(\frac{u-m(x)}{\sigma_{u}}\right)}^{2}]$$

(34) $${\underline{\mu }}_{A(x)}(u)=\lambda \cdot \exp\left[-\frac{1}{2}{\left(\frac{x-m(x)}{\sigma_{u}^{*}}\right)}^{2}\right]$$

(35) $$\mathrm{where}\;\sigma_{u}^{*}=\sigma_{u}\sqrt{\frac{\ln\left(l\right)}{\ln\left(\varepsilon \right)}},l=\left(l_{1}+l_{2}\right)/2$$

The fuzzy rules of the entire system can be written as follows:

$R^{i}$: If *x*_1_ is $J_{1}^{i}$ and …… *x_n_* is $J_{n}^{i}$ then u is $\tilde{P}$.

J and P are both type-two fuzzy sets, which have an interval consisting of two type-two fuzzy sets.

The fuzzy system can be rewritten as follows:

(36) H(t)=(σ+a)f(t)

(37) $$H(t)={\dot{u}}_{s}+Bu_{s}$$

(38) $$\sigma =\sigma_{1}+\sigma_{2}$$

where $f\left(t\right)$ is the output of the third type of fuzzy controller. It can be written as

(39) $$f(t)=h\left(C\left(t\right),\dot{C}\left(t\right)\right)$$

where $\hat{\sigma }$ is the estimate of the bounding factor, and we define the estimation error as

(40) $$\begin{array}{ll} e & =\hat{\sigma }-\sigma \\ & =\hat{\sigma }-(\sigma_{1}+\sigma_{2}) \end{array}$$

The estimate can be defined as

(41) $$\dot{\hat{\sigma }}=\left\{\begin{array}{ll} \frac{1}{\rho }\left|C\left(t\right)\right|\; & \left|C(t)\right|>l\\ 0 & \left|C(t)\right|\leq l \end{array}\right.$$

where ρ is the positive gain factor and l is the limit of $C\left(t\right)$.

Sliding mode control and PID control are introduced to design a robust uncertainty controller at the micro level by combining the interval fuzzy type-three system and the two of them to solve the uncertainty problem of micro–nano robots in liquid environments. Consider the following sliding mode surface:

(42) $$s_{0}=x_{2}+\Gamma x_{1}^{\left|\Omega_{1}\right|}+\Lambda x_{1}^{\left|\Omega_{2}\right|}$$

where $\Omega_{1}\Omega_{2}\in R$ are coefficients, respectively, and the first-order fast-terminal sliding-mode control variable is defined as:

(43) $${\dot{s}}_{0}={\dot{x}}_{2}+\Gamma \Omega_{1}{\left|x_{1}\right|}^{\Omega_{1}-1}{\dot{x}}_{1}+\Lambda \Omega_{2}{\left|x_{1}\right|}^{\Omega_{2}-1}{\dot{x}}_{1}$$

and $C\left(t\right)$ is

(44) $$C(t)=K_{p}s_{0}+K_{i}\int s_{0}dt+K_{d}\frac{ds_{0}}{dt}$$

Then,

(45) $$\begin{array}{l} C(t)=K_{p}s_{0}+K_{i}\int s_{0}dt+\\ \;K_{d}\left({\dot{x}}_{2}+\Gamma \Omega_{1}{\left|x_{1}\right|}^{\Omega_{1}-1}{\dot{x}}_{1}+\Lambda \Omega_{2}{\left|x_{1}\right|}^{\Omega_{2}-1}{\dot{x}}_{1}\right)\\ \;=K_{p}s_{0}+K_{i}\int s_{0}+\\ \;K_{d}F\left(x\right)+K_{d}G(x)u+K_{d}Ζ(x,t)+\\ \;K_{d}\left(\Gamma \Omega_{1}{\left|x_{1}\right|}^{\Omega_{1}-1}{\dot{x}}_{1}+\Lambda \Omega_{2}{\left|x_{1}\right|}^{\Omega_{2}-1}{\dot{x}}_{1}\;\right) \end{array}$$

F(x) and G(x) are unknown functions; Ζ(x,t) is a disturbance function. In order to obtain a better control error and a stable performance, the total control rate consists of two models, the equivalent control law and the switching law

(46) $$u=-K_{d}^{-1}G^{-1}(x)(u_{e}-K_{d}^{-1}u_{s})$$

where $u_{e}$ is

(47) $$\begin{array}{l} u_{e}=K_{p}s_{0}+K_{i}\int s_{0}+\\ \;K_{d}F\left(x\right)+K_{d}Ζ(x,t)+\\ \;K_{d}\left(\Gamma \Omega_{1}{\left|x_{1}\right|}^{\Omega_{1}-1}{\dot{x}}_{1}+\Lambda \Omega_{2}{\left|x_{1}\right|}^{\Omega_{2}-1}{\dot{x}}_{1}\;\right) \end{array}$$

and

(48) $${\dot{u}}_{s}+Bu_{s}=(\sigma_{1}+\sigma_{2}+A)sign(C(t))$$

where $u_{s}\left(0\right)=0$ and A, B are both small positive constants, and

(49) $$\left|K_{d}\sigma_{1}|\leq |\sigma_{2}\right|$$

**Assumption** **1.** *The interference function Z(x) in this paper is a bounded function, so it satisfies the following formula*:

$$\left|Z\left(x,t\right)\right|\leq \left\|\eta \right\|$$

(50) $$\frac{d(Z(x,t))}{dt}\leq \sigma$$

A microscopic liquid environment is more complex than a macroscopic environment, especially for acoustic robots. The existence of the sound field makes the entire system a high-uncertainty system. Using a type-two fuzzy system to estimate the errors and uncertainties in a dynamic system may not have enough degrees of freedom, so a type-three fuzzy system is introduced, which allows for the entire system to operate well in a complex external environment.

(51) $$\begin{array}{l} u_{s}\left(t\right)=(u_{s}\left(t_{0}\right)+\frac{1}{B}\left(\sigma_{1}+\sigma_{2}+A\right)h\left(C\left(t\right),\dot{C}\left(t\right)\right))e^{t-t_{0}}\\ -\frac{1}{B}\left(\sigma_{1}+\sigma_{2}+A\right)h\left(C\left(t\right),\dot{C}\left(t\right)\right) \end{array}$$

(52) $$\begin{array}{l} C(t)=K_{p}s_{0}+K_{i}\int s_{0}+\\ \;K_{d}F\left(x\right)+K_{d}G(x)u+K_{d}Ζ(x)+\\ \;K_{d}\left(\Gamma \Omega_{1}{\left|x_{1}\right|}^{\Omega_{1}-1}{\dot{x}}_{1}+\Lambda \Omega_{2}{\left|x_{1}\right|}^{\Omega_{2}-1}{\dot{x}}_{1}\;\right)\\ \;=u_{s}+\\ \;K_{d}\left(F\left(x\right)+K_{d}G(x)u+K_{d}Ζ(x,t)+\Gamma \Omega_{1}{\left|x_{1}\right|}^{\Omega_{1}-1}{\dot{x}}_{1}+\Lambda \Omega_{2}{\left|x_{1}\right|}^{\Omega_{2}-1}{\dot{x}}_{1}\right)\\ \;=u_{s}+K_{d}Z\left(x,t\right) \end{array}$$

and

$$\left|\sigma_{2}\right|\geq \left|K_{d}\sigma_{1}\right|\geq K_{d}{\left|u_{s}\right|}_{\max}\geq K_{d}\left|u_{s}\right|$$

and we have a derivative of the above formula

(53) $$\begin{array}{l} \dot{C}(t)={\dot{u}}_{s}+K_{d}Z\left(x,t\right)\\ \;=-Bu_{s}-(\sigma_{1}+\sigma_{2}+A)h\left(C\left(t\right),\dot{C}\left(t\right)\right)\\ \;+K_{d}\frac{dZ\left(x,t\right)}{dt} \end{array}$$

**Proof.**  Assume the following Lyapunov function:

(54) $$V=\frac{1}{2}C^{T}(t)C(t)+\frac{1}{2\rho }{\tilde{\sigma }}^{T}\tilde{\sigma }$$

and

(55) $$\begin{array}{l} \dot{V}=C^{T}(t)\dot{C}(t)+\frac{1}{\rho }e^{T}\dot{e}\\ \;=C^{T}(t)\dot{C}(t)+\frac{1}{\rho }\left(\hat{\sigma }-\sigma \right)\left(\dot{\tilde{\sigma }}-\dot{\sigma }\right)\\ \;=-C^{T}(t)Bu_{s}-C^{T}(t)(\sigma_{1}+\sigma_{2}+A)h\left(C\left(t\right),\dot{C}\left(t\right)\right)+\\ \;C^{T}(t)K_{d}\frac{dZ\left(x,t\right)}{dt}+\frac{1}{\rho }\left(\hat{\sigma }-\sigma \right)\dot{\tilde{\sigma }} \end{array}$$

We have

$$h\left(C\left(t\right),\dot{C}\left(t\right)\right)\leq 1$$

so

$$\left|C^{T}(t)\right|\left|h\left(C\left(t\right),\dot{C}\left(t\right)\right)\right|\leq C\left(t\right)$$

(56) $$\begin{array}{l} \dot{V}=C^{T}(t)\dot{C}(t)+\frac{1}{\rho }e^{T}\dot{e}\\ \;=-\left|C\left(t\right)\right|Bu_{s}-\left|C\left(t\right)\right|(\sigma_{1}+\sigma_{2}+A)+\\ \;\left|C\left(t\right)\right|K_{d}\frac{dZ\left(x,t\right)}{dt}+\frac{1}{\rho }\left(\hat{\sigma }-\sigma \right)\dot{\tilde{\sigma }}\\ \;=-\left|C\left(t\right)\right|Bu_{s}-\left|C\left(t\right)\right|(\sigma +A)+\\ \;\left|C\left(t\right)\right|K_{d}\frac{dZ\left(x,t\right)}{dt}+\frac{1}{\rho }\left(\hat{\sigma }-\left(\sigma_{1}+\sigma_{2}\right)\right)\rho \left|C\left(t\right)\right|\\ \;=-\left|C\left(t\right)\right|Bu_{s}-\left|C\left(t\right)\right|(\sigma +A)+\\ \;\left|C\left(t\right)\right|K_{d}\frac{dZ\left(x,t\right)}{dt}+\left(\hat{\sigma }-\left(\sigma_{1}+\sigma_{2}\right)\right)\left|C\left(t\right)\right|\\ \;=-\left|C\left(t\right)\right|bu_{s}+\left|C\left(t\right)\right|K_{d}\frac{dZ\left(x,t\right)}{dt}-\left|C\left(t\right)\right|\left(\sigma_{2}+\sigma_{1}+a\right)\\ \;\leq -a\left|C\left(t\right)\right|<0 \end{array}$$

Therefore, according to the above proof, the established system is asymptotically stable. □

## 3. Experiment

**Figure 1:**
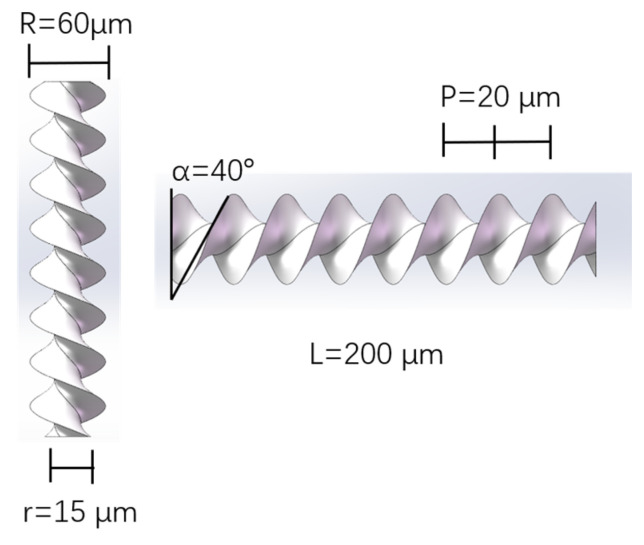
Micro–nano robot structure.

Inspired by the movement of spiral natural microorganisms (such as spirochetes), as shown in Figure 1, a spiral micro–nano robot was designed, consisting of a cylindrical core and a double-helical blade spiraling around the core. The double-helical blade can be seen as the superposition of two curved blades that spiral up along the surface of the cylindrical core. When this micro–nano robot is surrounded by fluid such as water and exposed to an acoustic field, it propels forward in a corkscrew-like motion, in which the particle rotates around the axis of its cylindrical core, and simultaneously translates along that axis. Micro–nano robots can be directly manufactured using high-resolution two-photon lithography 3D-printing technology. The micro robot manufactured in this study has a length of l=200 μm, an outer diameter of R=60 μm, and an inner diameter of r=15 μm. The robot is made of polymer material.

The system consists of two pairs of piezoelectric transducers with different parameters. The reason why there is a three-dimensional working space is that the micro–nano robot has a wall-tending property in a circular pipe. Driven by sound waves, it can move along any cavity wall. It can be seen that the motion control of the micro–nano robot in the three-dimensional working space can be divided into two independent directions. The position error of the micro–nano robot is the Euclidean distance of the three coordinates xyz. Finally, the path tracking of the micro–nano robot is achieved through the interaction of two piezoelectric transducers.

**Figure 2:**
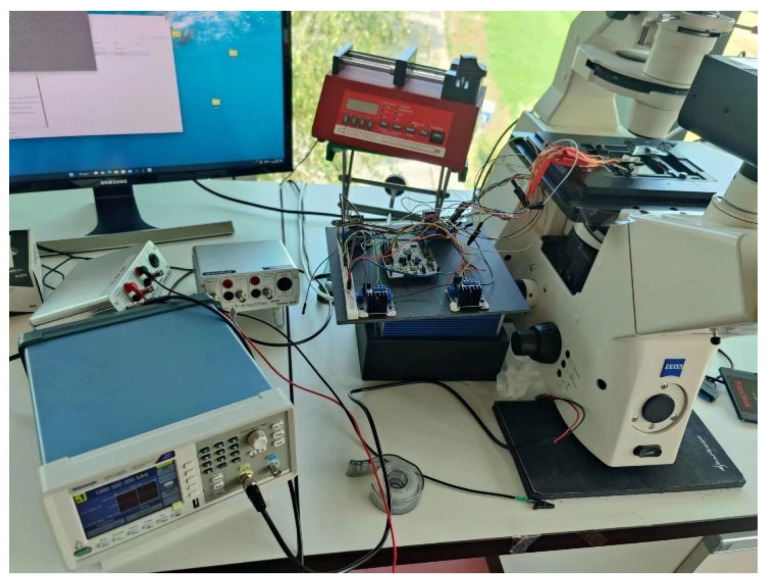
Experimental equipment.

The experimental setup is shown in Figure 2, which includes a signal generator, a camera, an inverted microscope, an STM32-based controller, a microfluidic channel, and a piezoelectric sensor (PZT, Steiner & Martins Inc., Davenport, FL, USA) used as an actuator, which are glued to the PDMS wall using two-component epoxy glue to fix them. The resonant frequencies of the ultrasonic piezoelectric transducers used are 2 MHz and 1 MHz. The function generator Tektronix Inc., Beaverton, OR, USA) and the microcontroller (STM32) connected to the camera (Kron Technologies Inc., Burnaby, BC, Canada) of the inverted microscope (AxioVertAxiovert 200, Carl Zeiss, Oberkochen, Germany) are connected by an internal Python code. The camera captures images at 33 frames per second, and these are processed by Python (3.11) for autonomous operation. In addition, the Python code controls the frequency, amplitude, and state of the piezoelectric sensor.

**Figure 3:**
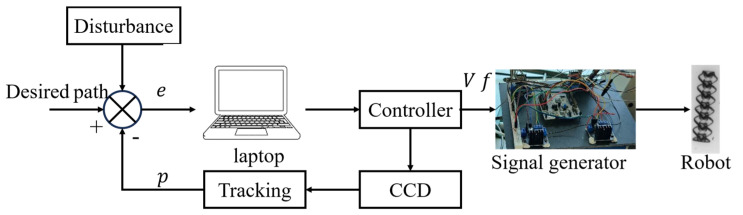
Experimental control logic.

The logic of the entire experiment is shown in Figure 3. This paper mainly designs a micro–nano robot designed for the purpose of delivering drugs in vivo, so the deep soft tissue mechanism is used as the application scenario. The robot needs to deliver the drug according to the specified path, so the micro–nano robot is placed in a cylindrical cavity for motion control experiments.

The micro–nano robot is given a path in advance, and then the positioning and tracking algorithm is used to capture the error between the current real-time position of the micro robot and the predetermined trajectory. The robust control algorithm based on the fuzzy system uses the error as the input to obtain the frequency and voltage of the control signal generator. In this way, the micro–nano robot is driven to move. At a specific frequency, the micro–nano robot rotates and moves forward. The voltage is generally set to the maximum value of the signal generator, which is determined by the physical distance between the robot and the signal generator and is positively correlated. The farther the distance is, the greater the voltage is. Then, the real-time position of the micro–nano robot is obtained using the positioning and tracking algorithm, and the next error is obtained, and the above operation is repeated.

In part, we studied the propulsion and navigation control experiments of micro–nano robots in response to acoustic stimulation in two-dimensional, and three-dimensional cavities. A cavity with a certain angle was created, which was divided into two-dimensional and three-dimensional cavities. The two-dimensional cavity is a straight line with a certain width, and the micro–nano robot can operate in the two-dimensional plane. The three-dimensional cavity is a cavity with a certain tilt angle.

**Figure 4:**
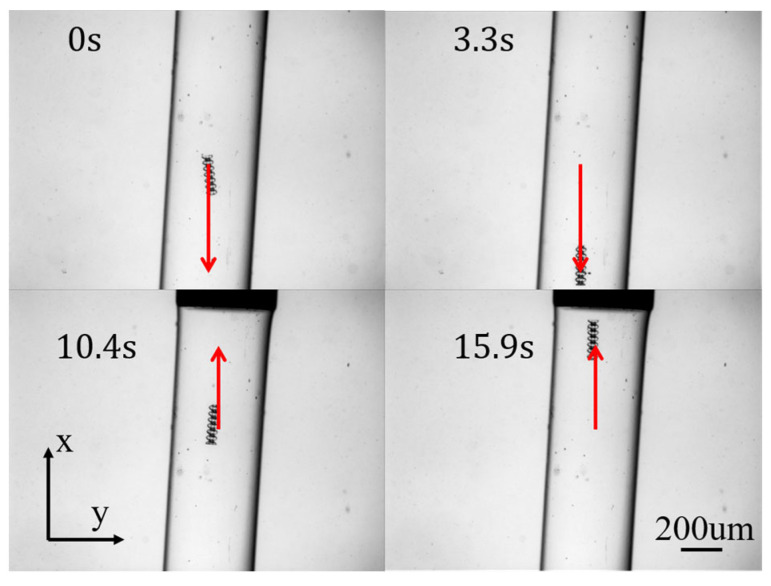
Robot one-dimensional motion.

**Figure 5:**
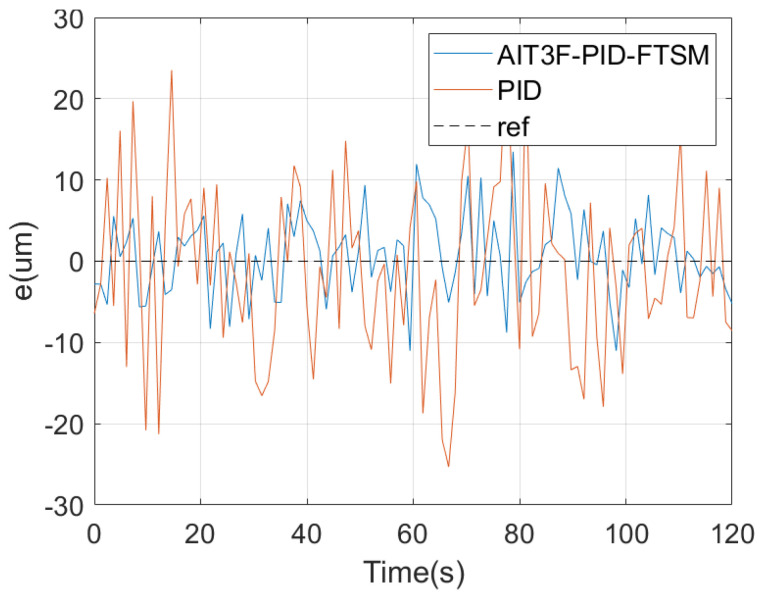
One-dimensional motion error of micro–nano robot.

In the first experiment, the reference path of the micro–nano robot was set to simulate one-dimensional motion in the vascular structure, that is the up-and-down motion curve, hereinafter referred to as path 1. Figure 4 shows the different stages of micro–nano robot tracking path 1. Figure 5 shows the one-dimensional path tracking error of the micro–nano robot.

**Figure 6:**
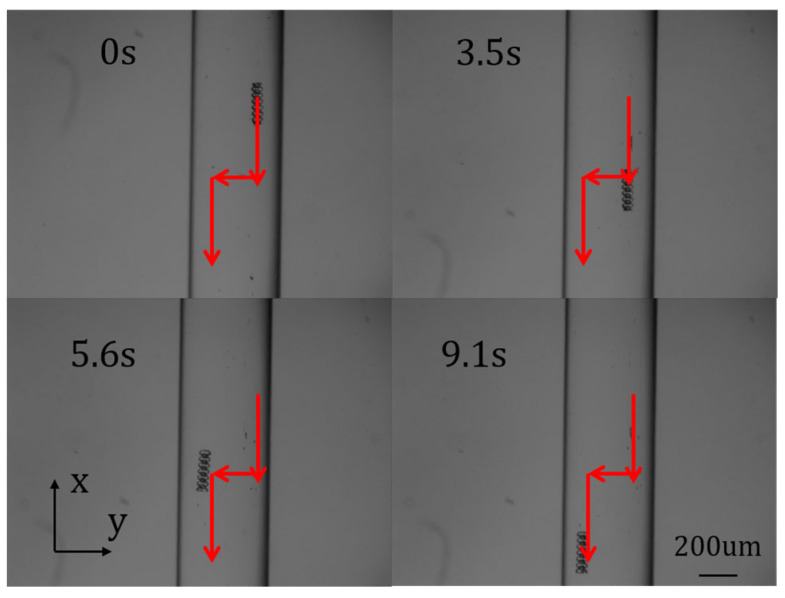
Robot two-dimensional motion.

**Figure 7:**
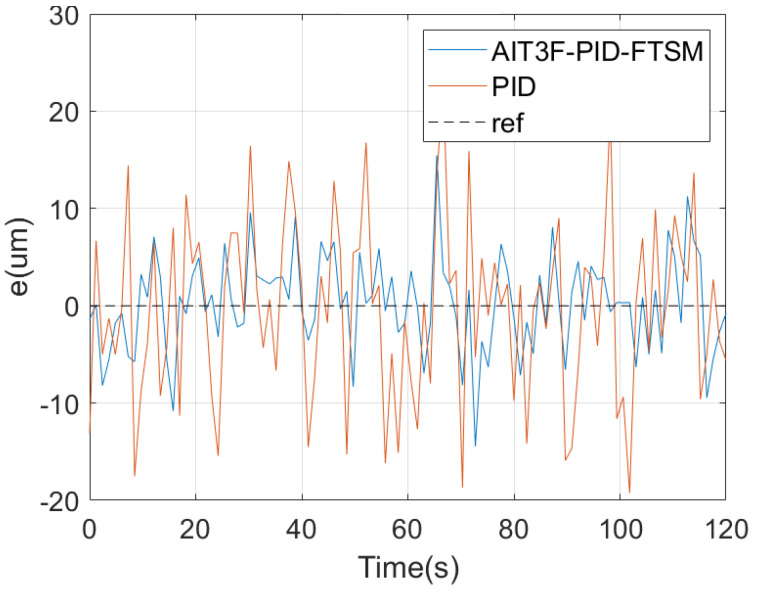
Two-dimensional motion error of micro–nano robot.

In the second experiment, the proposed control method was used to track a two-dimensional path, hereafter referred to as path 2. Figure 6 shows the different stages of micro–nano robot tracking path 2. The tracking error is shown in Figure 7.

**Figure 8:**
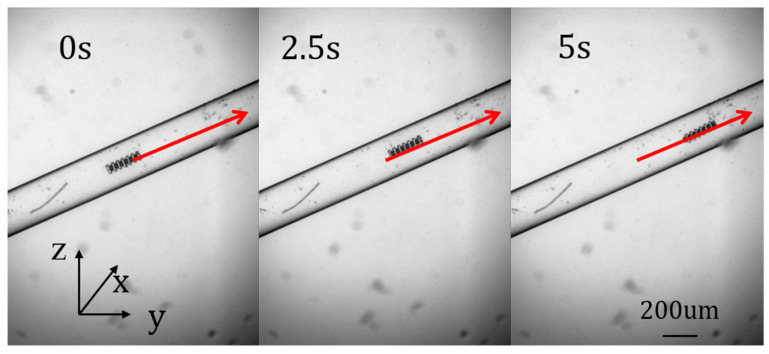
Robot three-dimensional motion.

**Figure 9:**
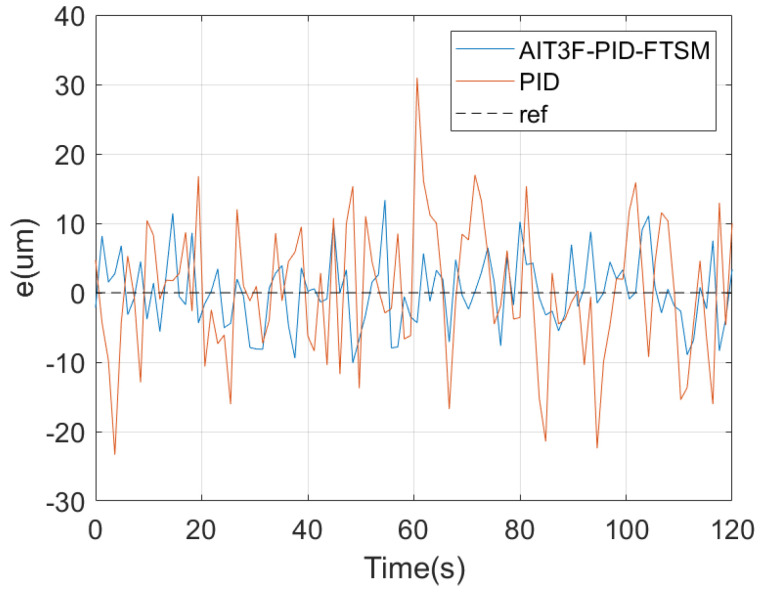
Three-dimensional motion error of micro–nano robot.

In the third experiment, a three-dimensional path was set up in the cavity of the simulated vascular structure for a robust control experiment on the micro–nano robot. The entire path is inclined upward at an angle, so the robot performed three-dimensional movement similar to climbing. Figure 8 shows the different stages of micro–nano robot tracking path 3. Figure 9 shows the tracking error. In summary, the robust control method of interval type-3 fuzzy fast-terminal sliding-mode control proposed in this paper can successfully realize the path tracking control of the micro–nano robot in the complex environment of a simulated vascular structure.

**Figure 10:**
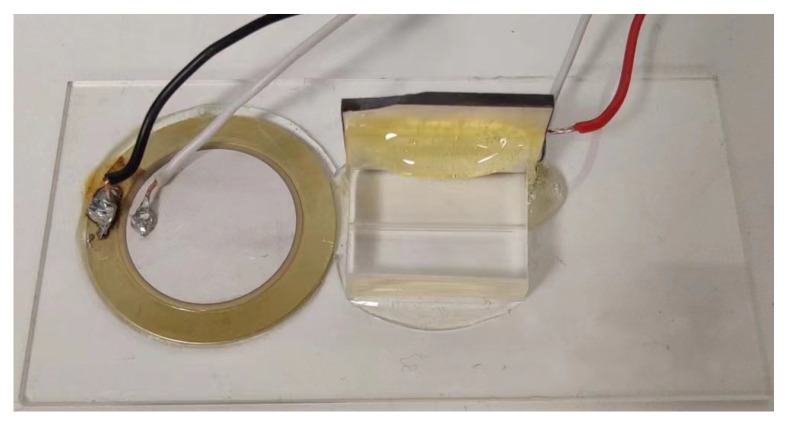
Overall experimental equipment diagram.

Under the action of the acoustic field, the micro robot moves unidirectionally along the channel at an approximately uniform speed, while rotating around its axis. Under the action of an acoustic field with a frequency of $f_{1}=12.53\;\mathrm{kHz}$ and the peak-to-peak voltage $60V_{PP}$, the micro robot moves to the left at a speed of 100 μm/s approximately and rotates clockwise at a speed of $1.66{\;s}^{-1}$. When the driving frequency of the acoustic signal is modulated, the micro robot shows counterclockwise rotation and translational motion in the opposite direction. The micro robot moves to the right at a speed of 80 μm/s and rotates at a speed of $0.91{\;s}^{-1}$. At $f_{1}$ startup, the micro robot translates along the microchannel at a speed of 100 μm/s. We turned off the acoustic signal, and the micro robot rolled back to the middle of the microchannel under the action of gravity. When the transducer was activated again at a frequency of $f_{2}$, the micro robot was pushed in the opposite direction at a speed of approximately 80 μm/s. The directionality of the micro robot can be changed by nearly 180° simply by changing the activation frequency of the acoustic signal. This unique feature has the potential to prevent blood vessels from being blocked when acoustically driven micro robots are used in medicine. The robot’s control area is shown in the Figure 10 below.

The robot’s left movement is translational, and it will not rotate around the axis. The principle of left movement is to use acoustic tweezers to capture the robot, and then move it by changing the stationary point of the standing wave. The camera shoots from the side, and the entire cavity forms a 15-degree angle with the ground. The maximum rotation speed of ω_z_ ≈ 1.66 Hz observed in the experiments results in a translational velocity of v_rtz_ ≈ 126 μm/s. We are still conducting experiments to compare the different driving methods. Because the micro–nano robot’s speed, voltage, and structure are all related, we are conducting experiments to find rules for the above factors. The diameter of the cavity is 500 μm.

Acoustic microstreaming is the primary driver of micro robot propulsion, and acoustic streaming is also considered a source of propulsion for other microparticles. One might wonder why acoustic streaming had a significant impact on our micro robot, despite its relatively large size. This can be explained by the unique shape of our particle. Acoustic streaming primarily occurs on fin-like blades, which have a length scale that is much smaller than that of the micro robot itself. Table 1 shows performance comparison of control group.

In all the experiments, rapid translational motion and rotation around the particle’s principal axis were observed. This strongly suggests that the geometry of the micro robot leads to coupling between translational and angular propulsion. Intuitively, this mechanism is induced by the hydrodynamic resistance of the micro robot’s helical fin structure.

Magnetically driven helical micro–nano robots have been widely used in low-Reynolds-number environments. Such micro–nano robots are usually designed with magnetic metal coatings and respond to rotating magnetic fields by magnetization. However, they are often limited by out-of-step frequency, which makes them unable to keep pace with the external magnetic field, and this limitation cannot be overcome by increasing the magnetic flux density because electromagnetic induction in medical applications needs to be maintained at a harmless level. In contrast, acoustically driven micro–nano robots exhibit a different propulsion mechanism that can achieve faster movement. Their translation speed is quadratic with the input voltage and has the potential to reach higher speeds. The parameter values are shown in the following Table 2.

## 4. Conclusions

This paper proposes an interval three-type fuzzy fast-terminal sliding-mode composite control method to solve the problem of having insufficient prior information on the dynamics of micro–nano robots. Compared with traditional robust control, this method has a higher degree of freedom and fault tolerance and does not require a complete understanding of uncertain dynamic functions or known disturbance bounds. This method effectively reduces chattering and proves system stability through the Lyapunov function. Comparative experiments with the classic control methods show that this method can significantly improve the trajectory tracking accuracy of micro–nano robots and reduce the tracking errors.

## Figures and Tables

**Table 1:** Performance comparison of control group.

Case		Case 1	Case 2	Case 3
Mean square error	PID	17.35 μm	18.34 μm	20.48 μm
	Proposed methods	12.82 μm	13.12 μm	14.23 μm

**Table 2:** Parameter values.

Name	Index	Value
Proposed methods	$K_{p}$, $K_{i}$, $K_{d}$, $\Omega_{1}$, $\Omega_{2}$, Γ, Λ, A, B, $\sigma_{1}$, $\sigma_{2}$, $l_{1}$, $l_{2}$, λ	250, 100, 0.01 0.5, 2.2, 1.09, 1.05 0.02, 0.5, 0.2, 0.5, 0.5, 0.5, 0.5

## Data Availability

The original contributions presented in the study are included in the article, further inquiries can be directed to the corresponding author.
